# Patterns of Care in Adjuvant Radiation Therapy for Stage II Endometrioid Endometrial Adenocarcinoma: A National Cancer Database Analysis

**DOI:** 10.1016/j.adro.2024.101698

**Published:** 2024-12-03

**Authors:** Jessica Cruttenden, Christopher Weil, Danae Byer, Lindsay Burt, Gita Suneja, David Gaffney, Cristina DeCesaris

**Affiliations:** a Department of Radiation Oncology, Huntsman Cancer Institute, University of Utah, Salt Lake City, Utah; b Division of Radiation Oncology, The University of Texas MD Anderson Cancer Center, Houston, Texas; c College of Medicine, Howard University, Washington, DC

## Abstract

**Purpose:**

Treating stage II endometrial cancer involves total hysterectomy, bilateral salpingo-oophorectomy, and risk-adapted adjuvant therapy. Professional guidelines support various adjuvant treatments, but high-level data supporting specific options are conflicting. We sought to evaluate adjuvant radiation therapy (RT) trends for these patients, hypothesizing increased utilization of pelvic external beam RT (EBRT) over time.

**Methods and Materials:**

Patients diagnosed in 2004-2019 with stage II endometrioid endometrial cancer who underwent total hysterectomy, bilateral salpingo-oophorectomy, and surgical staging were identified in the National Cancer Database. Patient characteristics per adjuvant RT received were compared using Wilcoxon rank sum and analysis of variance testing. Multivariable regression analysis (MVA) identified variables associated with EBRT, vaginal brachytherapy (VBT), or RT omission. A *P* value < .05 was significant, except in MVA, where Bonferroni correction was employed (*p* value < .017).

**Results:**

Patients meeting criteria totaled 18,798; 19% received adjuvant EBRT alone, 25% VBT alone, 24% EBRT + VBT, and 32% no RT. Adjuvant RT use increased from 2004 to 2019, particularly EBRT + VBT (*p* < .05). In MVA, community hospital treatment (odds ratio [OR], 1.8; *p* < .001), Midwest location (OR, 1.2; *p* = .02), single-agent chemotherapy receipt (OR, 6.9; *p* < .001), lymphovascular space invasion (OR, 1.4; *p* < .001), and positive surgical margins (OR, 1.8; *p* < .001) were positively associated with EBRT. No variables were positively associated with VBT. Black race (OR, 1.2; *p* = .03), community hospital treatment (OR, 1.4; *p* = .04), South (OR, 2.2; *p* < .001) or West (OR, 2.1; *p* < .001) location, distance >50 miles from the treatment center (OR, 1.5; *p* < .001), and grade 2 (OR, 1.2; *p* < .001) or 3 (OR, 1.3; *p* = .01) disease were associated with RT omission.

**Conclusions:**

Adjuvant RT for stage II endometrial cancer increased over time, particularly EBRT + VBT. Patient-related factors such as race, region, and distance from the treatment center were associated with RT omission, suggesting sociodemographic barriers to care. Tumor-related factors such as positive surgical margins and lymphovascular space invasion were associated with EBRT receipt, suggesting consideration of high-risk factors for locoregional recurrence in adjuvant RT approaches.

## Introduction

Endometrial cancer is the most common gynecologic malignancy in the United States.^1^ Stage II endometrial cancer, as defined by the 2009 International Federation of Gynecology and Obstetrics (FIGO) staging system, is characterized by cervical stromal invasion without extension beyond the uterus.^2^ The updated 2023 FIGO staging system has more specifically characterized nonaggressive histologic subtypes as stage IIA disease while broadening stage II disease to also include nonaggressive histologic subtypes with substantial lymphovascular space invasion (LVSI) (stage IIB) and aggressive histologic subtypes with any myometrial involvement (stage IIC).^3^ The standard of care for stage II endometrial cancer, as defined by the FIGO 2009 system, involves total hysterectomy along with bilateral salpingo-oophorectomy and surgical staging.^4^ However, recommendations for adjuvant therapy remain less well-defined and are often tailored to individual patients based on the presence or absence of high-risk features such as positive surgical margins and LVSI. The National Comprehensive Cancer Network (NCCN) provides several options for adjuvant therapies, including observation, vaginal cuff brachytherapy (VBT), pelvic external beam radiation therapy (EBRT), and chemotherapy.^4^

Multiple randomized controlled trials and meta-analyses comparing EBRT with observation in patients with early-stage endometrial cancer have demonstrated a reduction in locoregional recurrence (LRR) with EBRT.^5^, ^6^, ^7^, ^8^ Subsequently, given the propensity for recurrence at the vaginal cuff in early-stage disease, the Post Operative Radiation Therapy in Endometrial Carcinoma (PORTEC) 2 trial compared adjuvant VBT with EBRT and demonstrated comparable rates of LRR and survival between the 2 modalities.^9^^,^^10^ However, there are conflicting prospective data specifically looking at adjuvant therapy approaches for stage II disease as it has been defined in more recent decades.

The Gynecologic Oncology Group 249 trial randomized patients with FIGO stage I to II endometrial carcinoma to adjuvant VBT with chemotherapy or adjuvant EBRT alone.^11^ Patients in the VBT and chemotherapy arm had greater toxicity and higher rates of pelvic lymph node recurrence without a benefit in recurrence-free survival or overall survival.^11^ PORTEC 3 randomized patients with high-risk endometrial cancer to adjuvant EBRT with or without chemotherapy and failed to show survival benefits with chemotherapy in a subgroup analysis of stage I to II patients.^12^ Consensus guidelines by the American Society for Radiation Oncology (ASTRO), European SocieTy for Radiotherapy and Oncology, and NCCN thus recommend EBRT using intensity modulated radiation techniques for most cases of stage II endometrioid endometrial cancer with conditional recommendation for VBT boost when high-risk features are present, including close or positive surgical margins and LVSI.^4^^,^^13^^,^^14^ These same guidelines also allow for consideration of observation or VBT alone in select stage II patient populations.

Given the number of options within national guideline recommendations, adjuvant treatment approaches for these patients are often individualized, and practice patterns can be heterogeneous. This study aimed to use the National Cancer Database (NCDB) to examine patterns of adjuvant radiation therapy (RT) delivery in the management of stage II endometrial cancer, hypothesizing increased utilization of EBRT over time, particularly in conjunction with updated consensus guidelines.

## Methods and Materials

### Data source and study populations

The NCDB is a joint project of the Commission on Cancer of the American College of Surgeons and the American Cancer Society that consolidates deidentified information from multiple hospital cancer registries throughout the United States, including data on tumor characteristics, patient demographics, treatment approaches, and survival. The data used in this study were derived from a deidentified participant user file and are therefore exempt from institutional review board review.

### Patient selection

The NCDB was queried to identify all patients diagnosed in 2004-2019 with stage II endometrioid endometrial adenocarcinoma, as defined by the presence of cervical stromal invasion. Updated criteria for stage II disease per the 2023 FIGO staging system were not used for patient selection as they would not have been applied in the study period. Patients were excluded if they did not undergo total hysterectomy, bilateral salpingo-oophorectomy, and surgical staging with pelvic lymph node dissection or had absent adjuvant treatment variables, discordant staging data, metastatic disease, or high-risk histology, including carcinosarcoma, clear cell, or serous carcinoma.

### Statistical procedures

Clinical and sociodemographic differences were compared using Wilcoxon rank sum and analysis of variance testing. Multivariable logistic regression analysis (MVA) was used to identify variables that were predictive for receipt of adjuvant RT. A *p* value of <.05 was considered significant for all analyses, with the exception of MVA, where a Bonferroni correction was employed with a *p* value of <.017 was considered significant. Statistical analyses were conducted using STATA 18 (StataCorp LLC).

## Results

**Figure 1:**
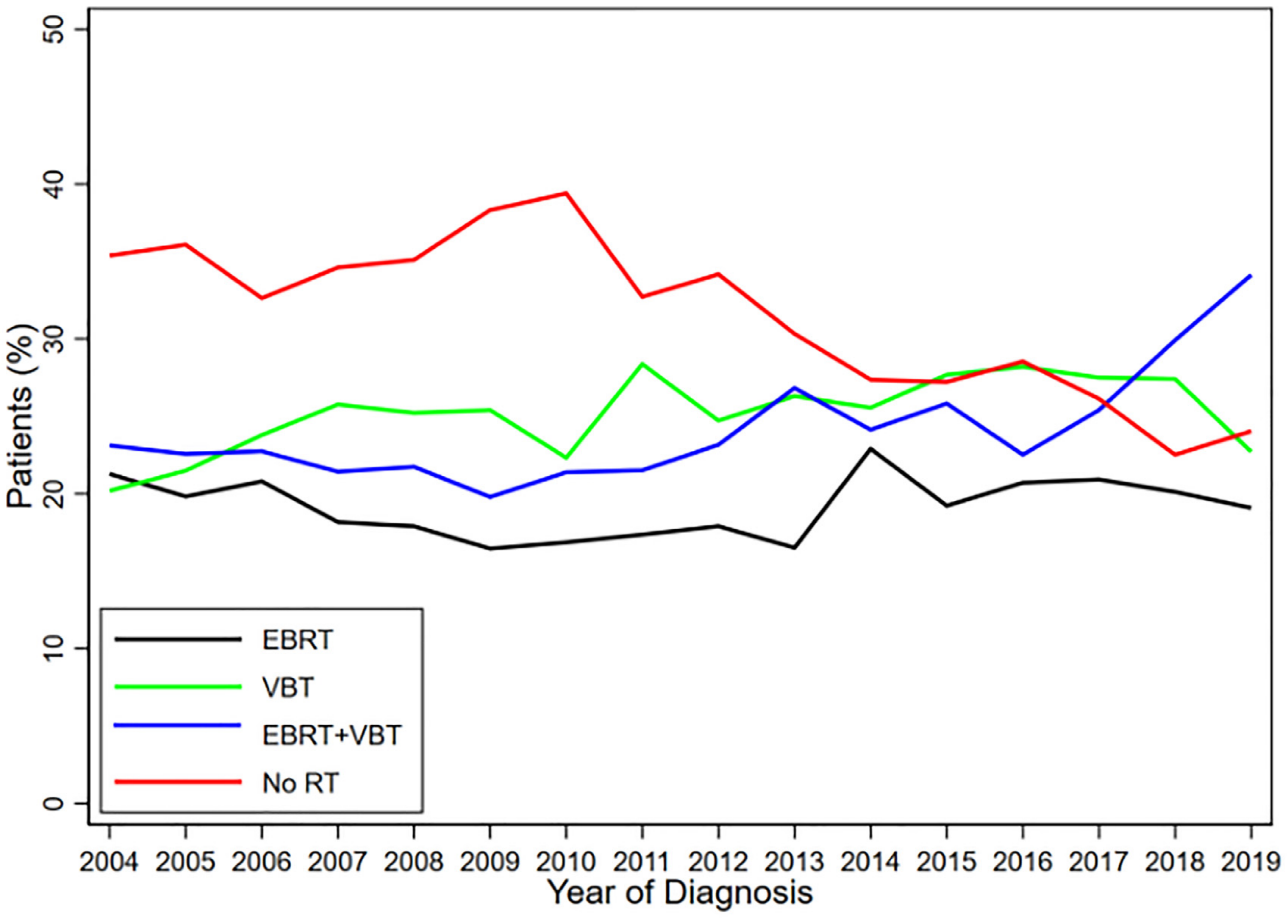
Use of all adjuvant radiation therapy (RT) approaches from 2004 to 2019. *Abbreviations:* EBRT = external beam radiation therapy; VBT = vaginal brachytherapy.

A total of 18,798 patients met the inclusion criteria. Patient sociodemographic and clinical characteristics are shown in Table 1. The median age was 60 years (IQR, 55-70 years). Most patients were White (85.8%), non-Hispanic (93.9%), insured (94.6%), and lived less than 50 miles from their treatment center (85.5%). Twelve percent of the study population received chemotherapy, and 68% of the study population received adjuvant RT. Of the patients who received RT, 28% received EBRT alone, 37% received VBT alone, and 35% received EBRT and VBT. Of the 2226 patients who received chemotherapy, 35% received chemotherapy alone, whereas 25% also received EBRT, 22% also received VBT, and 18% received EBRT and VBT. The use of all adjuvant RT approaches increased from 2004 to 2019, and the combination of EBRT with VBT was the most common treatment in 2019 (34% vs 23% in 2004, *p* < .05), as demonstrated in Fig. 1.

**Table 1:** Patient sociodemographic and clinical characteristics

RT group						
	EBRT only	VBT only	EBRT + VBT Boost	No RT	Total	*P* value
	n (%)	n (%)	n (%)	n (%)	N (%)	
Total	3590 (19.1)	4723 (25.1)	4510 (24.0)	5975 (31.8)	18,798 (100.0)	
Age group						<.001
18-60	1565 (43.6)	2159 (45.7)	2126 (47.1)	2501 (41.9)	8351 (44.4)	
61-99	2025 (56.4)	2564 (54.3)	2384 (52.9)	3474 (58.1)	10,447 (55.6)	
Race						.043
White	3075 (85.7)	4109 (87.0)	3874 (85.9)	5076 (85.0)	16,134 (85.8)	
Black	317 (8.8)	359 (7.6)	382 (8.5)	541 (9.1)	1599 (8.5)	
Asian	95 (2.6)	133 (2.8)	151 (3.3)	165 (2.8)	544 (2.9)	
Native American or Eskimo	16 (0.4)	18 (0.4)	11 (0.2)	32 (0.5)	77 (0.4)	
Native Hawaiian or Other Pacific Islander	6 (0.2)	12 (0.3)	9 (0.2)	12 (0.2)	39 (0.2)	
Other	81 (2.3)	92 (1.9)	83 (1.8)	149 (2.5)	405 (2.2)	
Ethnicity						.066
Non-Hispanic/unreported	3343 (93.7)	4457 (94.9)	4205 (93.9)	5548 (93.4)	17,553 (93.9)	
Hispanic-White	222 (6.2)	237 (5.0)	271 (6.1)	389 (6.5)	1119 (6.0)	
Hispanic-Black	4 (0.1)	3 (0.1)	3 (0.1)	5 (0.1)	15 (0.1)	
Percent of residents without a high school degree						
29%+	575 (18.0)	612 (14.9)	664 (17.0)	1053 (19.8)	2904 (17.6)	
20%-28.9%	758 (23.8)	944 (22.9)	884 (22.6)	1307 (24.5)	3893 (23.5)	
14%-19%	845 (26.5)	1041 (25.3)	1049 (26.8)	1274 (23.9)	4209 (25.4)	
<14%	1008 (31.6)	1522 (37.0)	1313 (33.6)	1693 (31.8)	5536 (33.5)	
Income						<.001
<$40,227	604 (18.8)	728 (17.4)	698 (17.6)	1163 (21.5)	3193 (19.1)	
$40,227-$50,353	771 (24.0)	906 (21.7)	891 (22.5)	1235 (22.9)	3803 (22.7)	
$50,354-$63,332	772 (24.0)	985 (23.6)	1014 (25.6)	1330 (24.6)	4101 (24.5)	
$63,333+	1072 (33.3)	1558 (37.3)	1355 (34.2)	1672 (31.0)	5657 (33.8)	
Insurance status						<.001
Uninsured	142 (4.0)	179 (3.8)	163 (3.6)	252 (4.2)	736 (3.9)	
Private	1618 (45.1)	2344 (49.6)	2247 (49.8)	2533 (42.4)	8742 (46.5)	
Medicaid	289 (8.1)	272 (5.8)	304 (6.7)	389 (6.5)	1254 (6.7)	
Medicare	1490 (41.5)	1868 (39.6)	1745 (38.7)	2710 (45.4)	7813 (41.6)	
Unknown	51 (1.4)	60 (1.3)	51 (1.1)	91 (1.5)	253 (1.3)	
Charlson-Deyo comorbidity index						.002
0	2665 (74.2)	3556 (75.3)	3370 (74.7)	4315 (72.2)	13,906 (74.0)	
1+	925 (25.8)	1167 (24.7)	1140 (25.3)	1660 (27.8)	4892 (26.0)	
Facility type						<.001
Academic	1222 (34.0)	1926 (40.8)	1591 (35.3)	2130 (35.6)	6869 (36.5)	
Comprehensive	2037 (56.7)	2551 (54.0)	2625 (58.2)	3414 (57.1)	10,627 (56.5)	
Community	231 (6.4)	82 (1.7)	177 (3.9)	234 (3.9)	724 (3.9)	
Unknown	100 (2.8)	164 (3.5)	117 (2.6)	197 (3.3)	578 (3.1)	
Location						.018
Metro	2839 (79.1)	3760 (79.6)	3648 (80.9)	4694 (78.6)	14,941 (79.5)	
Urban	581 (16.2)	701 (14.8)	656 (14.5)	923 (15.4)	2861 (15.2)	
Rural	62 (1.7)	94 (2.0)	71 (1.6)	116 (1.9)	343 (1.8)	
Unknown	108 (3.0)	168 (3.6)	135 (3.0)	242 (4.1)	653 (3.5)	
Geographic region						<.001
Northeast	827 (23.7)	1179 (25.9)	1135 (25.8)	963 (16.7)	4104 (22.5)	
Midwest	944 (27.0)	1347 (29.5)	1317 (30.0)	1215 (21.0)	4823 (26.5)	
South	1139 (32.6)	1365 (29.9)	1249 (28.4)	2529 (43.8)	6282 (34.5)	
West	580 (16.6)	668 (14.7)	692 (15.8)	1071 (18.5)	3011 (16.5)	
Distance from treatment facility						<.001
<50 miles	2884 (88.2)	3646 (85.9)	3540 (88.4)	4465 (81.5)	14,535 (85.5)	
≥50 miles	387 (11.8)	597 (14.1)	465 (11.6)	1014 (18.5)	2463 (14.5)	
Year of diagnosis						<.001
2004-2009	1506 (41.9)	1887 (40.0)	1733 (38.4)	2805 (46.9)	7931 (42.2)	
2010-2014	973 (27.1)	1355 (28.7)	1249 (27.7)	1743 (29.2)	5320 (28.3)	
2015-2019	1111 (30.9)	1481 (31.4)	1528 (33.9)	1427 (23.9)	5547 (29.5)	
Grade						<.001
1	850 (26.9)	1491 (35.8)	982 (25.8)	1701 (31.2)	5024 (30.3)	
2	1195 (37.9)	1531 (36.7)	1544 (40.6)	2191 (40.1)	6461 (39.0)	
3	677 (21.5)	576 (13.8)	791 (20.8)	892 (16.3)	2936 (17.7)	
Unknown	434 (13.8)	571 (13.7)	483 (12.7)	675 (12.4)	2163 (13.0)	
Chemotherapy						<.001
No chemo	3017 (84.0)	4136 (87.6)	3899 (86.5)	5376 (90.0)	16,428 (87.4)	
Multiagent	426 (11.9)	500 (10.6)	458 (10.2)	510 (8.5)	1894 (10.1)	
Single-agent	115 (3.2)	59 (1.2)	126 (2.8)	32 (0.5)	332 (1.8)	
Unspecified	32 (0.9)	28 (0.6)	27 (0.6)	57 (1.0)	144 (0.8)	
Chemotherapy timing						<.001
No chemotherapy/unknown	2546 (82.2)	3629 (85.9)	3366 (85.0)	4533 (88.6)	14,074 (85.8)	
Neoadjuvant	77 (2.5)	28 (0.7)	63 (1.6)	38 (0.7)	206 (1.3)	
Adjuvant	461 (14.9)	556 (13.2)	509 (12.8)	539 (10.5)	2065 (12.6)	
Neoadjuvant and adjuvant	13 (0.4)	10 (0.2)	24 (0.6)	8 (0.2)	55 (0.3)	
Myometrial invasion						<.001
<50%	71 (2.0)	126 (2.7)	52 (1.2)	271 (4.5)	520 (2.8)	
≥50%	72 (2.0)	78 (1.7)	65 (1.4)	141 (2.4)	356 (1.9)	
Unknown	3447 (96.0)	4519 (95.7)	4393 (97.4)	5563 (93.1)	17,922 (95.3)	
Lymphovascular space invasion						<.001
Not present	1216 (64.6)	2003 (75.7)	1574 (61.9)	1970 (69.7)	6763 (68.3)	
Present	666 (35.4)	644 (24.3)	967 (38.1)	857 (30.3)	3134 (31.7)	

*Abbreviations:* EBRT = external beam radiation therapy; RT = radiation therapy; VBT = vaginal brachytherapy.

On MVA, factors positively associated with receipt of EBRT included treatment at a community cancer center (odds ratio [OR], 1.8; 95% CI, 1.3-2.3) or comprehensive cancer center (OR, 1.1; 95% CI, 1.02-1.3), treatment in the Midwest (OR, 1.2; 95% CI, 1.03-1.4), receipt of single-agent chemotherapy (OR, 6.9; 95% CI, 2.9-16), presence of LVSI (OR, 1.4; 95% CI, 1.3-1.6), and positive surgical margins (OR, 1.8; 95% CI, 1.3-2.5). Conversely, treatment in the South (OR, 0.73; 95% CI, 0.63-0.85) or West (OR, 0.80; 95% CI, 0.67-0.95) and distance from a treatment center >50 miles (OR, 0.72; 95% CI, 0.61-0.85) were negatively associated with receipt of EBRT. Variables associated with receipt of EBRT are demonstrated in Table 2.

**Table 2:** Factors associated with receipt of external beam radiation therapy

	Predictors of receipt of any EBRT					
	Univariable			Multivariable		
	OR	95% CI	*P* value	OR	95% CI	*P* value
Age group						
18-60	-	-	-	-	-	-
61-99	0.79	0.76-0.83	<.001	0.96	0.84-1.1	.49
Race						
White	-	-	-	-	-	-
Black	1.03	0.93-1.1	.62	0.89	0.74-1.1	.25
Asian	1.1	0.92-1.3	.32	1.3	0.92-1.7	.16
Native American or Eskimo	0.71	0.45-1.1	.16	0.54	0.22-1.3	.18
Native Hawaiian or Other Pacific Islander	0.83	0.43-1.6	.56	0.87	0.30-2.5	.80
Other	0.90	0.74-1.1	.30	0.87	0.56-1.4	.54
Ethnicity						
Non-Hispanic/unreported	-	-	-	-	-	-
Hispanic-White	1.04	0.92-1.8	.49	0.95	0.76-1.2	.68
Hispanic-Black	1.2	0.42-3.2	.78	0.73	0.08-2.2	.31
Percent of residents without a high school degree						
29%+	-	-	-	-	-	-
20%-28.9%	0.98	0.89-1.1	.69	0.91	0.76-1.1	.27
14%-19%	1.1	0.99-1.2	.051	0.99	0.82-1.2	.89
<14%	0.97	0.89-1.1	.51	0.85	0.69-1.04	.11
Income						
<$40,227	-	-	-	-	-	-
$40,227-$50,353	1.1	1.02-1.2	.014	0.98	0.83-1.2	.83
$50,354-$63,332	1.1	1.02-1.2	.017	1.1	0.91-1.3	.36
$63,333+	1.1	0.99-1.2	.052	1.02	0.84-1.3	.83
Insurance status						
Uninsured	-	-	-	-	-	-
Private	1.1	0.96-1.3	.15	1.1	0.86-1.5	.42
Medicaid	1.3	1.1-1.5	.01	1.3	0.94-1.8	.11
Medicare	0.99	0.86-1.2	.99	0.95	0.72-1.3	.74
Unknown	0.95	0.71-1.3	.75	1.2	0.71-2.1	.45
Charlson-Deyo comorbidity index						
0	-	-	-	-	-	-
1+	0.95	0.89-1.02	.15	1.1	0.95-1.2	.30
Facility type						
Academic	-	-	-	-	-	-
Comprehensive	1.1	1.1-1.2	<.001	1.1	1.02-1.3	.02
Community	1.9	1.6-2.2	<.001	1.8	1.3-2.4	<.001
Location						
Metro	-	-	-	-	-	-
Urban	0.99	0.92-1.1	.86	1.1	1.00-1.4	.041
Rural	0.83	0.66-1.02	.09	0.87	0.60-1.3	.46
Unknown	0.77	0.66-0.91	.002	0.85	0.62-1.2	.31
Geographic region						
Northeast	-	-	-	-	-	-
Midwest	0.96	0.87-1.05	.38	1.2	1.02-1.4	.02
South	0.67	0.62-0.73	<.001	0.73	0.63-0.85	<.001
West	0.80	0.73-0.88	<.001	0.80	0.67-0.95	.009
Distance from treatment facility						
<50 miles	-	-	-	-	-	-
≥50 miles	0.67	0.59-0.76	<.001	0.72	0.61-0.85	<.001
Year of diagnosis						
2004-2009	-	-	-	-	-	-
2010-2014	0.96	0.90-1.03	.29	0.91	0.80-1.01	.16
2015-2019	1.3	1.2-1.4	<.001	1.1	0.96-1.2	.20
Grade						
1	-	-	-	-	-	-
2	1.3	1.2-1.4	<.001	1.1	0.99-1.3	.08
3	1.7	1.6-1.9	<.001	1.4	1.2-1.6	<.001
Unknown	1.3	1.2-1.4	<.001	1.1	0.98-1.3	.10
Chemotherapy						
No chemo	-	-	-	-	-	-
Multiagent	1.2	1.1-1.3	<.001	2.2	0.98-4.7	.055
Single-agent	3.6	2.9-4.6	<.001	6.9	2.9-16	<.001
Unspecified	0.95	0.68-1.3	.79	2.1	0.78-5.9	.14
Chemotherapy timing						
No chemotherapy/unknown	-	-	-	-	-	-
Neoadjuvant	2.9	2.2-3.9	<.001	0.83	0.34-2.0	.69
Adjuvant	1.2	1.1-1.3	<.001	0.45	.21-0.97	.043
Neoadjuvant and adjuvant	2.8	1.6-5.0	<.001	2.8	0.64-12	.17
Myometrial invasion						
<50%	-	-	-	-	-	-
≥50%	2	1.5-2.7	<.001	1.3	0.77-2.2	.32
Unknown	2.5	2.0-3.1	<.001	2.1	1.4-3.0	<.001
Lymphovascular space invasion						
Not present	-	-	-	-	-	-
Present	1.5	1.4-1.7	<.001	1.4	1.3-1.6	<.001
Surgical margins						
Negative	-	-	-	-	-	-
Positive	1.8	1.5-2.2	<.001	1.8	1.3-2.5	<.001

*Abbreviations:* EBRT = external beam radiation therapy; OR = odds ratio.

No factors were positively associated with the receipt of VBT. Age > 60 years (OR, 0.86; 95% CI, 0.76-0.98), treatment at a community cancer center (OR, 0.41; 95% CI, 0.31-0.54), distance from a treatment center >50 miles (OR, 0.72; 95% CI, 0.61-0.84), and grade 2 (OR, 0.86; 95% CI, 0.75-0.97) or 3 (OR, 0.80; 95% CI, 0.68-0.95) disease were negatively associated with receipt of VBT. Regionally, patients treated in the Midwest (OR, 0.84; 95% CI, 0.72-0.97), South (OR, 0.54; 95% CI, 0.47-0.63), or West (OR, 0.52; 95% CI, 0.44-0.62) were also less likely to have received VBT compared with those in the Northeast. Variables associated with receipt of VBT are demonstrated in Table 3.

**Table 3:** Factors associated with receipt of vaginal cuff brachytherapy

	Predictors of receipt of any VBT					
	Univariable			Multivariable		
	OR	95% CI	*P* value	OR	95% CI	*P* value
Age group						
18-60	-	-	-	-	-	-
61-99	0.85	0.81-0.90	<.001	0.86	0.76-0.98	.023
Race						
White	-	-	-	-	-	-
Black	0.88	0.80-0.98	.017	0.88	0.73-1.1	.17
Asian	1.1	0.94-1.3	.21	1.2	0.87-1.6	.29
Native American or Eskimo	0.62	0.39-0.98	.040	1.2	0.53-2.6	.70
Native Hawaiian or Other Pacific Islander	1.2	0.63-2.3	.59	2.6	0.85-7.7	.10
Other	0.78	0.64-0.95	.013	0.67	0.43-1.03	.07
Ethnicity						
Non-Hispanic/unreported	-	-	-	-	-	-
Hispanic-White	0.85	0.76-0.96	.010	0.98	0.78-1.2	.85
Hispanic-Black	0.68	0.24-1.9	.47	0.33	0.06-1.7	.18
Percent of residents without a high school degree						
29%+	-	-	-	-	-	-
20%-28.9%	1.1	1.02-1.2	.014	1.02	0.86-1.2	.79
14%-19%	1.3	1.1-1.4	<.001	0.96	0.80-1.2	.67
<14%	1.3	1.2-1.5	<.001	1.1	0.92-1.4	.24
Income						
<$40,227	-	-	-	-	-	-
$40,227-$50,353	1.1	1.01-1.2	.030	0.92	0.78-1.1	.34
$50,354-$63,332	1.2	1.1-1.3	.001	0.87	0.72-1.1	.11
$63,333+	1.3	1.2-1.4	<.001	0.91	0.74-1.1	.32
Insurance status						
Uninsured	-	-	-	-	-	-
Private	1.3	1.1-1.5	.002	1.01	0.78-1.3	.94
Medicaid	0.98	0.82-1.2	.82	0.84	0.62-1.1	.28
Medicare	0.99	0.85-1.2	.91	0.89	0.68-1.2	.39
Unknown	0.90	0.68-1.2	.48	0.83	0.48-1.4	.50
Charlson-Deyo comorbidity index						
0	-	-	-	-	-	-
1+	0.90	0.84-0.96	.001	0.89	0.80-1.00	.053
Facility type						
Academic	-	-	-	-	-	-
Comprehensive	0.91	0.85-0.96	.001	0.98	0.88-1.1	.02
Community	0.53	0.45-0.62	<.001	0.41	0.31-0.54	<.001
Location						
Metro	-	-	-	-	-	-
Urban	0.92	0.85-0.99	.035	1.04	0.89-1.2	.56
Rural	0.94	0.76-1.2	.59	1.4	1.00-2.1	.050
Unknown	0.88	0.75-1.03	.11	1.1	0.78-1.4	.72
Geographic region						
Northeast	-	-	-	-	-	-
Midwest	0.95	0.88-1.04	.28	0.84	0.72-0.97	.021
South	0.55	0.51-0.60	<.001	0.54	0.47-0.63	<.001
West	0.64	0.58-0.70	<.001	0.52	0.44-0.62	<.001
Distance from treatment facility						
<50 miles	-	-	-	-	-	-
≥50 miles	0.78	0.71-0.84	<.001	0.72	0.61-0.84	<.001
Year of diagnosis						
2004-2009	-	-	-	-	-	-
2010-2014	0.88	0.82-0.94	<.001	1.03	0.88-1.2	.09
2015-2019	1.2	1.1-1.3	<.001	1.2	1.04-1.3	.005
Grade						
1	-	-	-	-	-	-
2	0.94	0.87-1.0	.08	0.86	0.75-0.97	.017
3	0.90	0.82-0.98	.022	0.80	0.47-0.63	.009
Unknown	0.98	0.89-1.1	.70	0.89	0.440.62	.13
Chemotherapy						
No chemo	-	-	-	-	-	-
Multiagent	1.1	0.97-1.2	.17	0.79	0.38-1.6	.53
Single-agent	1.3	1.1-1.6	.014	0.81	0.37-1.8	.61
Unspecified	0.65	0.46-0.90	.011	0.73	0.28-1.9	.52
Chemotherapy timing						
No chemotherapy/unknown	-	-	-	-	-	-
Neoadjuvant	0.80	0.61-1.1	.12	0.83	0.36-1.9	.67
Adjuvant	1.1	0.98-1.2	.11	1.5	0.71-3.0	.31
Neoadjuvant and adjuvant	1.6	0.95-2.8	.08	1.7	0.54-5.3	.37
Myometrial invasion						
<50%	-	-	-	-	-	-
≥50%	1.3	0.98-1.7	.07	1.7	0.93-2.4	.10
Unknown	1.9	1.6-2.3	<.001	3.3	1.3-2.5	.001
Lymphovascular space invasion						
Not present	-	-	-	-	-	-
Present	0.94	0.87-1.03	.17	0.91	0.81-1.02	.11
Surgical margins						
Negative	-	-	-	-	-	-
Positive	0.84	0.71-1.01	.058	0.83	0.61-1.1	.24

*Abbreviations:* OR = odds ratio; VBT = vaginal brachytherapy.

Omission of any adjuvant RT was positively associated with Black race (OR, 1.2; 95% CI, 1.02-1.5), treatment at a community cancer center (OR, 1.4; 95% CI, 1.01-1.8), treatment in the South (OR, 2.2; 95% CI, 1.9-2.6) or West (OR, 2.1; 95% CI, 1.8-2.6), distance from a treatment center >50 miles (OR, 1.5; 95% CI, 1.3-1.8), and grade 2 (OR, 1.2; 95% CI, 1.1-1.4) or 3 (OR, 1.3; 95% CI, 1.1-1.5) disease. In contrast, receipt of single-agent chemotherapy (OR, 0.14; 95% CI, 0.05-0.38), >50% myometrial invasion (OR, 0.58; 95% CI, 0.31-0.60), and positive surgical margins (OR, 0.66; 95% CI, 0.46-0.96) were negatively associated with adjuvant RT omission. Variables associated with RT omission are shown in Table 4.

**Table 4:** Factors associated with radiation omission

	Factors associated with radiation omission					
	Univariable			Multivariable		
	OR	95% CI	*P* value	OR	95% CI	*P* value
Age group						
18-60	-	-	-	-	-	-
61-99	1.2	1.1-1.2	<.001	1.1	0.94-1.2	.26
Race						
White	-	-	-	-	-	-
Black	1.1	0.99-1.2	.05	1.2	1.02-1.5	.030
Asian	0.95	0.78-1.1	.58	0.94	0.67-1.3	.74
Native American or Eskimo	1.5	0.98-2.4	.059	1.00	0.43-2.3	.99
Native Hawaiian or Other Pacific Islander	0.97	0.49-1.9	.93	0.68	0.21-2.2	.52
Other	1.3	1.03-1.6	.023	1.7	1.1-2.7	.019
Ethnicity						
Non-Hispanic/unreported	-	-	-	-	-	-
Hispanic-White	1.2	1.02-1.3	.028	1.04	0.82-1.3	.73
Hispanic-Black	1.1	0.37-3.2	.89	2.1	0.49-28.8	.32
Percent of residents without a high school degree						
29%+	-	-	-	-	-	-
20%-28.9%	0.89	0.80-0.98	.021	0.96	0.80-1.2	.68
14%-19%	0.76	0.69-0.84	<.001	0.92	0.75-1.1	.39
<14%	0.77	0.70-0.85	<.001	0.90	0.73-1.2	.35
Income						
<$40,227	-	-	-	-	-	-
$40,227-$50,353	0.84	0.76-0.93	.001	1.1	0.92-1.3	.32
$50,354-$63,332	0.84	0.76-0.92	<.001	1.1	0.93-1.4	.23
$63,333+	0.73	0.69-0.80	<.001	1.1	0.88-1.3	.45
Insurance status						
Uninsured	-	-	-	-	-	-
Private	0.78	0.69-0.92	.003	0.98	0.74-1.3	.89
Medicaid	0.86	0.71-1.1	.14	1.01	0.73-1.4	.92
Medicare	1.02	0.87-1.2	.81	1.2	0.91-1.6	.18
Unknown	1.1	0.80-1.5	.62	1.1	0.62-2.0	.71
Charlson-Deyo comorbidity index						
0	-	-	-	-	-	-
1+	1.1	1.1-1.2	<.001	1.03	0.91-1.2	.65
Facility type						
Academic	-	-	-	-	-	-
Comprehensive	1.1	0.99-1.1	.11	1.03	0.91-1.2	.64
Community	1.1	0.90-2.3	.47	1.3	1.01-1.8	.039
Location						
Metro	-	-	-	-	-	-
Urban	1.04	1.0-1.2	.86	0.88	0.74-1.1	.17
Rural	1.1	0.89-1.4	.09	0.77	0.52-1.1	.20
Unknown	1.3	1.1-1.5	.002	1.3	0.91-1.7	.16
Geographic region						
Northeast	-	-	-	-	-	-
Midwest	1.1	0.99-1.2	.058	1.1	0.93-1.3	.26
South	2.2	2.0-2.4	<.001	2.2	1.9-2.6	<.001
West	1.8	1.6-2.0	<.001	2.1	1.7-2.5	<.001
Distance from treatment facility						
<50 miles	-	-	-	-	-	-
≥50 miles	1.6	1.4-1.7	<.001	1.5	1.3-1.8	<.001
Year of diagnosis						
2004-2009	-	-	-	-	-	-
2010-2014	1.1	1.04-1.2	.002	0.90	0.80-1.02	.056
2015-2019	0.71	0.65-0.77	<.001	0.77	0.69-0.86	<.001
Grade						
1	-	-	-	-	-	-
2	1.0	0.93-1.1	.95	1.2	1.1-1.4	.004
3	0.85	0.78-0.94	.001	1.3	1.1-1.5	.011
Unknown	0.89	0.80-0.99	.028	1.2	0.98-1.4	.079
Chemotherapy						
No chemo	-	-	-	-	-	-
Multiagent	0.76	0.68-0.84	<.001	0.57	0.26-1.2	.15
Single-agent	0.22	0.15-0.32	<.001	0.14	0.05-0.38	<.001
Unspecified	1.35	0.96-1.9	.79	0.79	0.28-2.2	.65
Chemotherapy timing						
No chemotherapy/unknown	-	-	-	-	-	-
Neoadjuvant	0.48	0.33-0.67	<.001	1.2	0.14-3.1	.69
Adjuvant	0.74	0.67-0.83	<.001	1.2	0.57-2.6	.62
Neoadjuvant and adjuvant	0.36	0.17-0.76	.007	0.49	0.09-2.6	.40
Myometrial invasion						
<50%	-	-	-	-	-	-
≥50%	0.60	0.46-0.80	<.001	0.58	0.36-0.94	.027
Unknown	0.41	0.34-0.49	<.001	0.43	0.31-0.60	<.001
Lymphovascular space invasion						
Not present	-	-	-	-	-	-
Present	0.92	0.83-1.0	.07	0.97	0.86-1.1	.60
Surgical margins						
Negative	-	-	-	-	-	-
Positive	0.71	0.58-0.86	.001	0.66	0.46-0.96	.029

*Abbreviations:* OR = odds ratio.

## Discussion

Conflicting prospective data exist to guide adjuvant therapy approaches for stage II endometrial carcinoma. Though professional consensus guidelines are supportive of EBRT to decrease LRR risk for most stage II patients, practice patterns may be heterogeneous and individualized based on the presence or absence of clinical risk factors. This study demonstrates that the use of adjuvant RT in the treatment of patients with stage II endometrioid endometrial carcinoma has increased over time, particularly the use of EBRT with VBT boost (*p* < .05).

Tumor-related factors such as positive surgical margins, LVSI, and myometrial invasion were associated with the utilization of EBRT, suggesting concordance with professional guidelines and consideration of high-risk factors for LRR in adjuvant RT approaches. Contemporary to the 2004-2019 study period, the prognostic importance of substantial LVSI (≥4 vessels) and molecular classification has been established by pooled analyses of PORTEC 1 and PORTEC 2.^15^^,^^16^ As such, stage II endometrial cancer has been broadened by the 2023 FIGO staging system to include nonaggressive histologies with substantial LVSI (stage IIB), aggressive histologies with myometrial invasion (stage IIC), and p53 abnormal cancers of any histology confined to the uterus with any myometrial invasion (stage IICm_p53abn_).^3^ The practice patterns in this study reflect consideration of these very risk factors, as EBRT was significantly more likely to be used in patients with any LVSI, and RT was significantly less likely to be omitted in patients with >50% myometrial invasion.

Patient-related sociodemographic factors such as Black race, geographic region, and farther distance from the treatment center were associated with the omission of adjuvant RT entirely. Though disparities in care access and delivery are well-documented in the literature, prior analyses have demonstrated that Black women with endometrial cancer are similarly or more likely to receive adjuvant RT despite being less likely to receive indicated surgeries or chemotherapies.^17^, ^18^, ^19^, ^20^, ^21^ Of particular note, however, Black women are more likely to be diagnosed with aggressive, nonendometrioid histologies, which were excluded from this study population.^17^ Regardless, this data could be suggestive of sociodemographic inequities in the delivery of adjuvant RT for this patient population. Patterns of adjuvant RT use also varied geographically, particularly the use of VBT, which may be reflective of the national distribution of brachytherapy equipment.^22^

This study is limited in its retrospective design and utilization of a database with coding inaccuracies, missing data, and a population mostly reflective of White, insured, nonrural patients treated at academic or comprehensive cancer centers, thus limiting the generalizability. Consideration of these weaknesses in our methodology, however, contributes to this study's strengths with the exclusion of patients with missing data while still maintaining a large sample size reflective of patients from multiple institutions across the nation.

This study is further limited by the absence of certain variables within the NCDB, which may otherwise guide adjuvant treatment decision-making. For example, NCCN and ASTRO guidelines support the use of adjuvant VBT alone for microscopic cervical stromal involvement when additional risk factors are absent, but the extent of cervical stromal invasion is not coded for within the NCDB.^4^^,^^13^ Retrospective analyses of PORTEC 1, PORTEC 2, and PORTEC 3 have also demonstrated value in using molecular classification to predict adjuvant therapy response in endometrial cancer, and both the NCCN and ASTRO support the use of molecular testing for patients considering adjuvant therapy.^4^^,^^13^^,^^16^^,^^23^ Though these molecular factors are not coded for within the NCDB, decision-making based on molecular testing may take time to adopt outside of a clinical trial. This testing is also not yet ubiquitous, thus limiting its utilization in adjuvant treatment decision-making for centers in less-resourced settings.

## Conclusions

Adjuvant RT utilization in patients with stage II endometrial carcinoma has increased over time, particularly when high-risk factors are present. The role of adjuvant RT in treating these patients will only become more important as a greater number of patients with high-risk pathologic and molecular features will be classified as stage II under the updated 2023 FIGO staging system. Adjuvant RT practices will also continue to evolve as predictive molecular testing becomes more widely used. Continued evaluation of adjuvant treatment patterns in light of this new staging system and molecular testing will be critical in addressing continued challenges in treatment decision-making and delivery. Ongoing research, education, and guidelines dissemination are necessary to optimize appropriate and equitable delivery of adjuvant RT.
